# Draft genome sequence of strain Khilg15.8, a member of a potential new genus in the family *Idiomarinaceae*

**DOI:** 10.1128/mra.00160-26

**Published:** 2026-06-04

**Authors:** Ekaterina B. Kudryashova, Elena V. Ariskina, Sergey V. Tarlachkov, Elena S. Kashkak, Dulma D. Tsyrenova, Elena Y. Abidueva

**Affiliations:** 1 G. K. Skryabin Institute of Biochemistry and Physiology of Microorganisms, All-Russian Collection of Microorganisms, Federal Research Center, Pushchino Scientific Center for Biological Research of the Russian Academy of Sciences 133901 , Pushchino, Russia; 2 Tuvan State University 292184, Kyzyl, Russia; 3 Institute of General and Experimental Biology, Siberian Branch, Russian Academy of Sciences 230761 , Ulan-Ude, Russia; Indiana University, Bloomington, Bloomington, Indiana, USA

**Keywords:** draft genome, haloalkaliphilic microorganism, alkaline-saline lake

## Abstract

Here we report the genomic sequence of the strain Khilg15.8, isolated from the alkaline-saline Lake Khilganta (Zabaykalsky Krai, Russia), characterized by sharp seasonal and annual fluctuations in mineralization and pH of the water. These genomic data suggest classifying the strain into a new genus and species in the family *Idiomarinaceae*.

## ANNOUNCEMENT

Lake Khilganta is one of the many saline and soda-saline lakes in the Zabaykalsky Krai (1), with high water mineralization and alkaline, unlike others. The lake is characterized by sharp fluctuations in water salinity and alkalinity, depending on the weather (2). During dry periods, the water has been observed drying up to puddles. Strain Khilg15.8 was isolated from a bottom sediment sample collected in September 2018 from Lake Khilganta (50.848611–115.123889) with water salinity and alkalinity values of 32 g/L and 9.78 g/L, respectively, in period sampling. Pre-isolation, 5 g of sample was shaken in 100 mL of sterile seawater (5% sea salt, wt/vol) supplemented with 0.5% (wt/vol) Na_2_CO_3_ at 180 rpm for 2 h at 24°C, aerobically. Serial 10-fold dilutions were plated on the 7-fold diluted nutrient medium #1 GRM agar (Obolensk, Russia) supplemented with 5% sea salt and adjusted to pH 9.5 with 1 M sodium sesquicarbonate and incubated aerobically at 24°C for 30 days. Single colonies were selected and purified by the streak-plating method onto #1 GRM agar with 5% sea salt and pH 9.5 plates. The plates were incubated aerobically at 28°C. One of the isolates obtained in pure culture was designated Khilg15.8. Initially, the phylogenetically closest species were determined on the EzBioCloud server (http://www.ezbiocloud.net [3]) by analyzing its 16S rRNA gene sequence, obtained using Sanger sequencing (accession number PZ153134). The Khilg15.8 is most closely related to *Aliidiomarina* spp. (97%–98.3%), with *Aliidiomarina soli* Y4G10-17(T) showing the highest level of similarity (98.3%). The above values were below the cutoff line for defining novel species (98.7% [4]). Here we present a draft genome of Khilg15.8, allowing a more accurate taxonomic placement. This genomic resource can serve as the basis for studying the biotechnological potential of this microorganism, as well as its role in the biogeochemical processes of Lake Khilganta.

A pure culture of Khilg15.8 was inoculated into GRM-broth medium with 5% sea salt and pH 9.5 and cultivated at 28°C for 2 days on a shaker, then was pelleted by centrifugation. Genomic DNA was isolated using a DNeasy PowerSoil Pro Kit (Qiagen, Hilden, Germany). The shotgun library was prepared using an NEBNext Ultra II DNA Library Prep Kit (New England Biolabs, Ipswich, MA, USA) and sequenced on an Illumina MiSeq instrument in paired read mode (2 × 300 nt). Default parameters were used for all software unless otherwise noted. The quality of the reads was checked with FastQC v0.12.1 (5). Adapter sequences and low-quality regions in raw reads were cut with Trimmomatic v0.39 (6) using the following options: ILLUMINACLIP:TruSeq3-PE-2.fa:2:30:10; SLIDINGWINDOW:4:15; and MINLEN:30. Clean reads after trimming were assembled using SPAdes v4.1.0 (7) with options --cov-cutoff, auto; and --careful. Genome coverage was assessed with QUAST v5.3.0 (8). The annotation was performed at the NCBI Prokaryotic Genome Annotation Pipeline v6.10 (9).

A total of 1,766,300 paired-end reads with a length of 301 bp (531.7 Mb) were obtained from the sequencing. As a result, clean 1,403,337 reads with an average length of 208 bp (291.9 Mb) were assembled into 26 scaffolds with a 104-fold coverage. Scaffold N50 is 1,562,648 bp, and the largest scaffold is 1,562,648 bp. The genome assembly size is 2,810,438 bp with an average G + C content of 53.2%. A total of 2,622 protein-coding genes (255 of which are coding hypothetical proteins), 60 tRNAs, 17 complete or partial rRNAs, and 4 ncRNAs were predicted.

Genome-based taxonomic determination using the Microbial Genomes Atlas (10) revealed that Khilg15.8 represents a new genus and species in the family *Idiomarinaceae*. These results differed from taxonomic inferences based on 16S rRNA gene sequence analysis. The pairwise similarity values of the ANI (https://gitlab.pasteur.fr/GIPhy/OGRI) and dDDH (11) (≤67.7% and ≤20.4%, respectively) were significantly lower than generally accepted thresholds for species delimitation (ANI, 95%, and dDDH, 70% [12]). The values of the recommended index for delimitation of genera AAI (https://gitlab.pasteur.fr/GIPhy/OGRI) between Khilg15.8 and the type species of the genera of the family *Idiomarinaceae* (≤63.1%) were below the threshold values for delimitation of genera in different bacterial groups (60%–80% [13]). The findings are important for studies in the area of bacterial systematics and taxonomy.

## Data Availability

The raw reads have been deposited to the NCBI SRA under accession no. SRR34722807, and the whole-genome shotgun project has been deposited in DDBJ/ENA/GenBank under accession no. JBPCGR000000000. The version described in this paper is the first version, JBPCGR010000000.
